# An optimized 4C-seq protocol based on cistrome and epigenome data in the mouse RAW264.7 macrophage cell line

**DOI:** 10.1016/j.xpro.2022.101338

**Published:** 2022-04-19

**Authors:** Zhiqiang Huang, Cheng Wang, Eckardt Treuter, Rongrong Fan

**Affiliations:** 1 Department of Biosciences and Nutrition, Karolinska Institutet, NEO, 14183 Huddinge, Sweden; 2 Smurfit Institute of Genetics, Trinity College Dublin, Dublin 2, Ireland

**Keywords:** Bioinformatics, Sequence analysis, Cell Biology, Cell culture, Genomics, Sequencing, Molecular Biology

## Abstract

Chromosome conformation capture combined with high-throughput sequencing (4C-seq) is a powerful tool to map genomic DNA regions that communicate with a specific locus of interest such as functional single-nucleotide polymorphism (SNPs)-containing regions. This protocol describes detailed steps to perform 4C-seq in mouse macrophage RAW264.7 cells, starting from the primer design based on cistrome and epigenome data, sample processing, and to the bioinformatics analysis.

For complete details on the use and execution of this protocol, please refer to Huang et al. (2021).

## Highlights

• 4C-seq in mouse RAW264.7 macrophage cells
• Applicable to any region with a proven or suspected regulatory role in transcription
• Integrated cistrome and epigenome data to refine the primer design of 4C-seq protocol

## Graphical abstract

**Figure:**
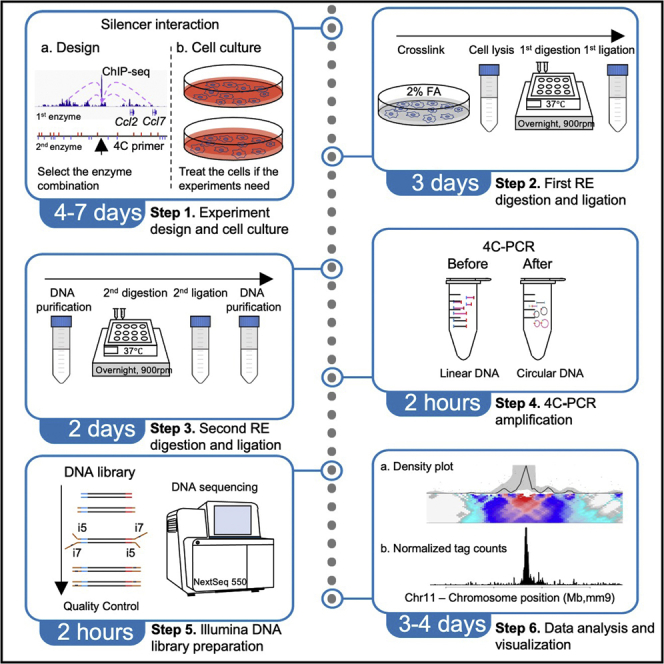

## Before you begin

Transcription is tightly regulated by chromatin remodeling events (Li et al., 2007). The dynamic interplay of transcription factors and coregulators in distal and proximal genomic *cis*-regulatory regions (enhancers, silencers, promoters) is crucial for this process, as it controls the physical interaction of those regions through looping structures (Kolovos et al., 2012; Maksimenko and Georgiev, 2014; Segert et al., 2021). To identify the transcriptional regulatory targets of specific enhancers and silencers, it is essential to map the promoters or transcription start sites (TSSs), which they physically contact. Chromosome conformation capture (3C) is a technology that was initially developed for studying such interactions (Dekker, 2006). This PCR-based technique is mainly used to validate the looping between two genomic elements, i.e., a candidate distal enhancer with an adjacent gene promoter (Hagege et al., 2007). However, the fast development of functional genetics and epigenetics requires more detailed and unbiased genome structure mapping techniques. Firstly, because the tissue-specific enhancer/silencer regions not only communicate with promoters but also with each other to form regulatory clusters. Secondly, because the direct targets of enhancers and silencers are not necessarily the closest gene promoters, which limits the application of the 3C technology (Chen et al., 2021; Han et al., 2018; Lindsay et al., 2018; McCord et al., 2020). To overcome this, 4C (or circular 3C) has been developed. The general idea was to introduce a secondary enzymatic digestion and ligation procedure (van de Werken et al., 2012). By amplifying the ligated library with PCR primers in the ‘bait’ element (also called ‘viewpoint’), it is possible to determine the sequences of all genomic regions that contact the bait through next generation sequencing (NGS). The technique was further developed into 5C, ChIA-PET, Hi-C, and promoter capture Hi-C (Belton et al., 2012; Dekker, 2006; Li et al., 2017; Martin et al., 2015; van Berkum and Dekker, 2009; van de Werken et al., 2012). As these genome-wide methods and their analysis are relatively advanced and expensive, the 4C-seq method provides an alternative for many standard laboratories to determine high-resolution DNA-DNA interaction profiles within specific genomic loci. This allows to investigate the looping dynamics within topologically associating domains (TADs) defining the expression of genes and gene clusters as well as to explore the functional aspects of disease-relevant single nucleotide polymorphisms (SNPs) from genome-wide association studies (GWAS) (Meddens et al., 2016; van de Werken et al., 2012).

In this protocol, we describe the 4C-seq method in detail using RAW264.7 cells, a mouse macrophage cell line widely used to study acute and metabolic inflammation. We specifically outline how cistrome and epigenome data can be integrated in the primer designing step, critical for the entire protocol. Because intra-TAD chromatin loops are facilitated by transcription factors and coregulators, many of which are co-localized in open chromatin regions, the binding centers of those factors can be obtained by chromatin immunoprecipitation sequencing (ChIP-seq). The binding sites of these factors reflect the coherent loci of the chromatin loops, and therefore can be used as references to improve the accuracy of the 4C primers. We specifically describe the 4C protocol using examples of recently identified *Ccl2* enhancer and silencer as a bait (Huang et al., 2021).

## Key resources table

**Table:** 

REAGENT or RESOURCE	SOURCE	IDENTIFIER
**Chemicals, peptides, and recombinant proteins**		

DpnII	NEB	Cat# R0543M
NlaIII	NEB	Cat# R0125L
1 kb DNA ladder	Thermo Fisher	Cat# 10787018
Agarose	Thermo Fisher	Cat# R0492
AMPure beads	Beckman Coulter	Cat# A63881
ATP	NEB	Cat# P0756S
DMEM, high glucose pyruvate	Thermo Fisher	Cat# 41966052
DNA loading buffer	Thermo Fisher	Cat# R0611
dNTP	Thermo Fisher	Cat# 18427089
EDTA	Sigma	Cat# 1233508
Ethanol	VWR	Cat# 20821.310
Fetal bovine serum (FBS)	Thermo Fisher	Cat# A3160802
Formaldehyde	Sigma	Cat# F8775
Hepes	Sigma	Cat# H3375
IGEPA CA-630	Sigma	Cat# I8896
LPS	Sigma	Cat# F8666
IL4	Sigma	Cat# SRP3211
Penicillin-streptomycin	Thermo Fisher	Cat# 15070063
Phenol: Chloroform: Isoamyl Alcohol 25:24:1	Sigma	Cat# P2069
Potassium chloride (KCl)	Sigma	Cat# P9333
Potassium phosphate monobasic (KH_2_PO4)	Sigma	Cat# P0662
Protease inhibitors	Sigma	Cat# 5056489001
Protease K	Thermo Fisher	Cat# EO0492
RNase A	Thermo Fisher	Cat# EN0551
Sodium acetate (NaA_C_)	Sigma	Cat# S2889
Sodium chloride (NaCl)	Sigma	Cat# S7653
Sodium phosphate dibasic (Na_2_HPO4)	Sigma	Cat# 255793
T4 ligase	NEB	Cat# M0202L
T4 ligation buffer	NEB	Cat# B0202S
TAE buffer	Thermo Fisher	Cat# B49
Triton X-100	Sigma	Cat# X100
Water (DNase and RNase free)	Thermo Fisher	Cat# 10977035

**Critical commercial assays**		

Expand long template PCR system	Roche	Cat# 117590600001
QIAquick PCR purification kit	QIAGEN	Cat# 28104
ChargeSwitch PCR Clean-Up Kit	Thermo Fisher	Cat# CS12000
Qubit Fluorometric Quantification kit	Thermo Fisher	Cat# Q33238
SMARTer DNA unique dual index kits	Takara	Cat# R400660-R400663
SMARTer PicoPLEX library preparation kit	Takara	Cat# R400676
NEXTFLEX Rapid DNA-Seq Library Prep Kit	PerkinElmer	Cat# NOVA-5144-01
NEBNext Ultra II DNA Library Prep Kit	NEB	Cat# E7103
DNeasy Blood & Tissue Kits	QIAGEN	Cat# 69504

**Deposited data**		

NGS data for this study	This paper	GSE195943
Zenodo reference	This paper	10.5281/zenodo.6393191
GPS2 ChIP-seq data	GEO	GSM4848601

**Experimentalmodels: Celllines**		

RAW264.7	ATCC	Cat# TIB-71 RRID: CVCL_0493

**Oligonucleotides**		

4C-seq primers for Ccl2 silencer bait (S) Reading primer: GTAAAAGTGATTAGAAGAGA**GATC** Inverse primer: GACTGCACTCACCATCA**CATG**	This paper	N/A

**Software and algorithms**		

4Cseqpipe	https://github.com/changegene/4Cseqpipe	N/A
Bioconductor	http://www.bioconductor.org/	RRID: SCR_006442
FastQC	http://www.bioinformatics.babraham.ac.uk/projects/fastqc/	RRID: SCR_014583
Bowtie2	http://bowtie-bio.sourceforge.net/index.shtml	RRID: SCR_005476
GraphPad Prism	https://www.graphpad.com/scientific-software/prism/	RRID: SCR_002798
HOMER	http://biowhat.ucsd.edu/homer/index.html	RRID: SCR_010881
pipe4C-master	https://github.com/deLaatLab/pipe4C	N/A
SnapGene	https://www.snapgene.com/	RRID: SCR_015052
Galaxy	https://usegalaxy.org/	RRID: SCR_006281
RStudio	http://www.rstudio.com/	RRID: SCR_000432
Samtools	http://htslib.org/	RRID: SCR_002105

IRanges	http://www.bioconductor.org/packages/2.13/bioc/html/IRanges.html	RRID: SCR_006420
ShortRead	http://www.bioconductor.org/packages/2.11/bioc/html/ShortRead.html	RRID: SCR_006813

**Other**		

1.5 mL tube	Sigma	Cat# T6649
50 mL tube	Sarstedt	Cat# 62.547.254
T75 Flasks	Sarstedt	Cat# 83.3911.002
T175 Flasks	Sarstedt	Cat# 83.3912.002
150 × 25 mm Cell Culture Dishes	VWR	Cat# 734-0013
96 well cell plates	Sigma	Cat# CLS3596
Cell lifter	Sigma	Cat# CLS3008
NanoDrop 2000/2000c Spectrophotometers	Thermo Fisher	Cat# ND-2000
−20°C freezer	Ninolux	Cat# LCexv 4010
−80°C freezer	Thermo Fisher	Cat# 15152373
Cell counter	Thermo Fisher	Cat# AMQAX1000
Centrifuge	Eppendorf	Cat# 5804R
Countess Cell Counting Chamber Slides	Thermo Fisher	Cat# C10228
DNA gel visualization system	Bio-Rad	Cat# 170-8126
Magnetic Stand-96	Thermo Fisher	Cat# AM10027
Microcentrifuge	Thermo Fisher	Cat# 75002410
PCR Machine	Applied Biosystems	Cat# 3342342
Pump	VACUSAFE	Cat# 158 300
Qubit 4 Fluorometer	Thermo Fisher	Cat# Q33238
TapeStation system 4200	Agilent	Cat# G2991AA
Thermomixer	Eppendorf	Cat# 5382000015

## Materials and equipment

Dissolve one protease inhibitor tablet in 1 mL distilled water for 50× solution and store it at −20°C.

5 M NaCl: Dissolve 292.2 g NaCl in 800 mL distilled water. Adjust the final volume with water to 1 L and store the buffer between 20°C to 25°C.

0.5 M EDTA: Dissolve 146.12 g EDTA in 800 mL distilled water. Adjust the final volume with water to 1 L and store the buffer between 20°C to 25°C.

1 M Hepes-KOH, pH 7.5: Dissolve 238.3 g Hepes 800 mL distilled water. Adjust the pH to 7.5 using 1 M KOH. Adjust the final volume with distilled water to 1 L and store the buffer between 20°C to 25°C.

**Table:** 10% FBS DMEM medium (4°C for storage and 37°C pre-warm for usage)

Reagent	Final concentration	Amount
DMEM	N/A	445 mL
Fetal bovine serum (FBS)	10%	50 mL
Penicillin-streptomycin	50 U	5 mL
**Total**	**N/A**	**500 mL**

**Table:** Dulbecco’s Phosphate Buffered Saline (DPBS) (keep on ice till use)

Reagent	Final concentration	Amount
NaCl	137.93 mM	8.06 g
KCl	2.67 mM	0.2 g
Na_2_HPO_4_	8.06 mM	1.14 g
KH_2_PO_4_	1.47	0.2 g
ddH2O	N/A	N/A
**Total**	**N/A**	**1,000 mL**

**Table:** Cell lysis buffer (keep on ice till use)

Reagent	Final concentration	Amount
NaCl	140 mM	560 μL from 5 M stock
EDTA	1 mM	40 μL from 0.5 M stock
IGEPA CA-630	0.5%	100 μL
Triton X-100	1%	200 μL
Hepes-KOH, pH 7.5	50 mM	1 mL from 1 M stock
Protease inhibitors	1×	400 μL from 50× stock
ddH_2_O	N/A	17.7 mL
**Total**	**N/A**	**20 mL**

**Table:** 2% Formaldehyde solution (fresh prepared, leave it between 20°C to 25°C till use)

Reagent	Final concentration	Amount
Formaldehyde	2%	2.7 mL from 37% stock
DPBS	N/A	47.3 mL
**Total**	**N/A**	**50 mL**

***Note:*** Freshly prepared DMEM medium is recommended as macrophages are very sensitive to any potential irritant. DPBS buffer can be stored between 20°C to 25°C. Freshly prepared Cell lysis buffer is recommended. EDTA, NaCl, Hepes-KOH stock buffer are kept between 20°C to 25°C. The rest of chemicals and reagents are normally stored between 20°C to 25°C. All other reagents in this protocol are listed in the key resources table.

**CRITICAL:** The formaldehyde is harmful, so it is better to open and take the formaldehyde solution in ventilation hood or any suitable devices.

## Step-by-step method details

### 4C primer design

**Figure 1:**
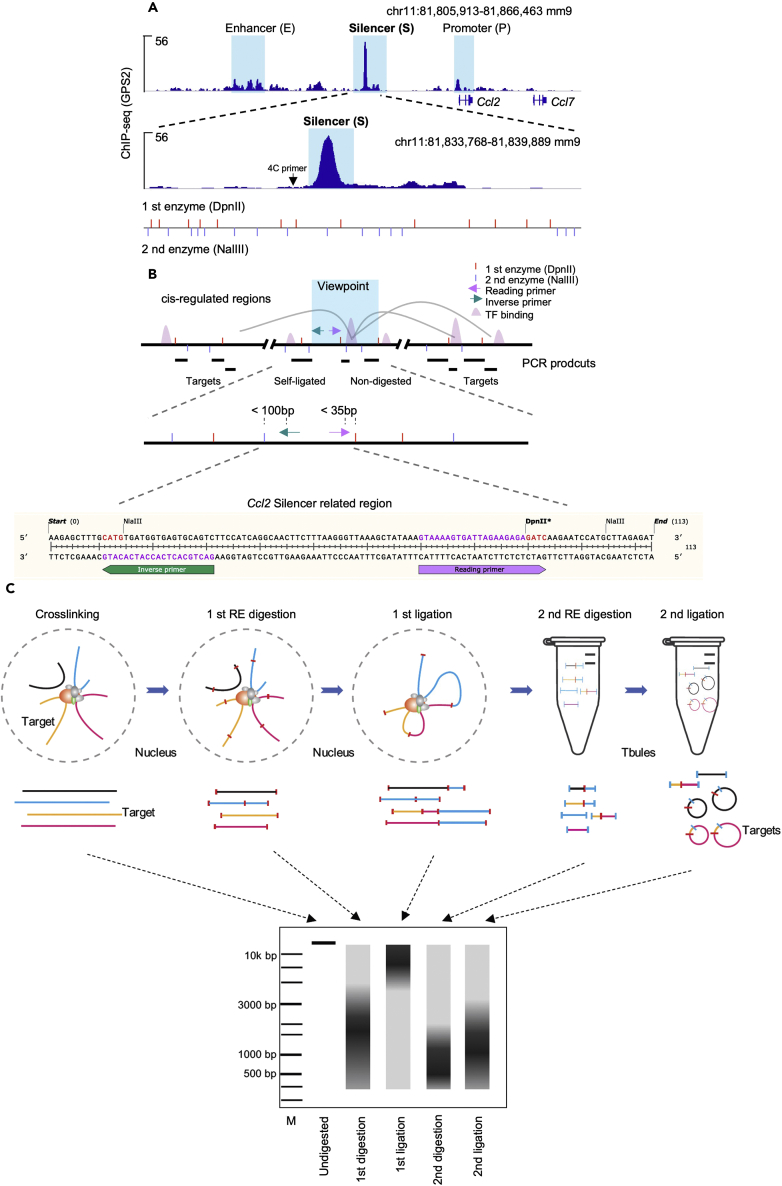
A guide for the designing of a 4C-seq primer (A) One example showing the integration with epigenetic profile within a gene locus (GPS2 ChIP-seq GEO: GSM4848601) to mark the local DNA environment. *Ccl2* silencer and promoter and the nearby enhancer regions are highlighted. The silencer region is further zoomed in to check the enzyme distribution (DpnII and NalIII are used in this case). (B) Schematic representation of 4C-PCR primers design principles. The interaction fragments are amplified with a reading primer tails. The “self-ligation” and “non-digestion” fragments are the top list of the amplified products. The viewpoint window is further zoomed in to highlight the PCR primers. Normally, the reading primer is within 35 bp to the first enzyme site and inverse PCR primer is within 100 bp to the second enzyme site. (C) Schematic representation of the 4C DNA fragments formation workflow in each step and the integration with the DNA gel distribution.

Primer design is the crucial step for the success of 4C-seq. Cistrome or epigenome data are helpful to define the open chromatin regions enriched with transcription factors (TFs) and coregulators. Such regions usually define the 3D chromatin structures that aim to be detected by 4C-seq. We can also get the profile of the local environment (Figure 1A) in the gene locus of interest. Restriction sites within and surrounding the center of those regions can be analyzed using SnapGene or other relevant software. The chromatin region we select in this protocol was identified as a silencer in *Ccl2* locus previously (Huang et al., 2021). The DNA profiles interacting with this specific silencer might be helpful for understanding its transcriptional regulatory mechanisms.

NlaIII, DpnII, Csp6I, BfaI and MboI (4-cutter) are commonly used as the step 1 restriction enzymes since they are widely distributed in the target genome. They are also highly efficient in digesting the crosslinked DNA. The first restriction enzyme site must avoid the transcription factor binding centers because those regions usually facilitate the loop formation and disruption of them may affect the 4C-seq efficiency (Figure 1B). Once a proper first restriction enzyme is selected, the reading primer (inverse PCR) is fixed which contains the restriction site sequence in the 3′ terminal (in this case, we used DpnII). The reading primer is normally between 20 to 35 bp close to the first enzyme site. The inverse PCR primer is dependent on the location and can be selected in different restriction enzyme combinations. In this case, we used DpnII-NalIII for detecting the mice *Ccl2* Silencer interactions in RAW cells. The inverse PCR primer should be close to the second enzyme in the target region and there should be no additional second enzyme sites between the reading primer and inverse primer. The inverse PCR primer is normally 20 bp, but can be up to 100 bp to the closest second enzyme site (Figure 1B).

Illustrated step strategy is shown in Figure 1C. The 4C-PCR will amplify all the possible interaction fragments which contain the reading primer. The detailed protocol is shown below.

### Amplify the RAW264.7 macrophage cell line

**Timing: 4–7 days for amplifying and seeding the RAW264.7 cells**

**Figure 2:**
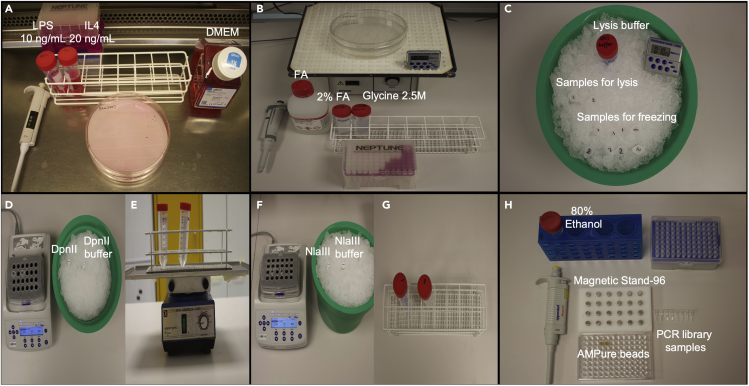
Preparation of 4C template and library preparation for sequencing

All the workflows are summarized in Figure 1C as an overview of a 4C experiment and the main experimental stages are shown in Figure 2.

1. Thaw the RAW264.7 cells and culture them in 75 cm flask, 37°C, 5% CO_2_. The cells are maintained in DMEM medium supplemented with 10% heat inactivated FBS and 1% Penicillin/ streptomycin (P/S).
2. Change the medium the next day and check the cell states.
3. When the cells become to 90% confluency in the 75 cm flask, use the cell lifter to scrap out the RAW264.7 cells and passage them in another two 150 cm plates with 1:3 dilution ratio. Wait for another two days until the cells fully recover.
4. When the cells become 90% confluent, scrap out the cells and count the cell number. Seed 2 × 10^7^ cells/plate in four 150 cm plates with 30 mL full DMEM medium. Maintain the cells overnight and wait for another day.

**CRITICAL:** RAW264.7 cells are very sensitive to any endotoxin (e.g., lipopolysaccharide or LPS) and cytokines (e.g., interleukin 6 or IL6) in the culture medium. The normal RAW264.7 cells are small, round shaped with tiny antennas. The cells are mildly attached to the flask in the normal condition. When the cells are activated by inflammatory stimulus during the amplification, they will firmly attach to the flask and the cell shape will change from round to spindle.

### Macrophages activation for the 4C-seq experiment

**Timing: 2 h for LPS/IL4 treatment, 10 min for crosslinking cells**

The treatment is optional according to experiment requirements. In this protocol, we used the WT RAW264.7 cells without any treatment (start from step 7).

5. Prepare 10 ng/mL LPS or 20 ng/mL IL4 using the full DMEM culture medium (optional) (Figure 2A).
6. Set two plates of the cells as control group and change the medium with 25 mL 10% FBS DMEM medium. Set the other two plates as treatment group and change the medium to 25 mL 10% FBS DMEM medium supplemented with 10 ng/mL LPS or 20 ng/mL IL4 cells for two hours (optional).
7. Wash the cells with 10 mL DPBS at room temperature two times. Prepare 2% formaldehyde (stock: 37% formaldehyde) solution with DPBS.
8. Crosslink the protein-DNA complexes by incubating the cells with 10 mL 2% formaldehyde for 10 min at room temperature with gentle shaking.
9. Stop the cross-linking by adding glycine into the formaldehyde fixed cells to a final concentration of 125 mM (stock: 2.5 M glycine) and incubate for 5 min at room temperature with gentle shaking (Figure 2B).
10. Wash the cells three times with ice cold DPBS.
11. Scrap the plates in 10 mL cold DPBS (with 1× protease inhibitors) and centrifuge at 1,000 × *g* for 5 min to pellet the cells.
  a. Wash two more times with DPBS (with protease inhibitors).
  b. Count the crosslinked cells and aliquot 10^7^ cells into 1.5 mL tubes.
12. Spin down the cells at 1,000 × *g* for 5 min at 4°C. Remove the supernatant and freeze the samples at −80°C or continue with cell lysis.

∗ SAFE Stop point. The cell pellets can be stored at −80°C for a few months.

**CRITICAL:** The viability is important to get high quality and stable results. More than 90% of alive cells are good cell viability to process the downstream experiments. Less than 70% of alive cells might change the data reproducibility. Normally, 10^5^–10^7^ cell numbers are a suitable range, which are dependent on the gene loci (high or low expression), region of interest (enhancer or silencer), and the formation time of the DNA looping, etc (Krijger et al., 2020). The strong looping anchors give more signals than the weak ones. Low input cell numbers might not be able to capture low or moderate chromatin interactions. So, the minimum cell number can be 10^5^ if the condition does not permit, but people must understand that the low cell number will restrict the 4C contact building. For inducible inflammatory genes, the intra-TAD DNA interactions are dynamic, and triggered by stimulus such as LPS, IL4, etc. Local eRNA expression can reflect the potential loop facilitated *cis*-regulatory element activities and can be used for selecting the best monitoring time of the treatment. The crosslinking condition (both formaldehyde concentration and treatment time) can be applied to the other *in vitro* cells including primary cells and cell lines. For 3D organoids or tissue samples, the crosslinking condition should be optimized before the further steps.

### 4C-library preparation

**Timing: 7–9 days**

The following steps are the cell lysis for 4C template. We perform the analysis in biological replicates. Here we only describe the protocol in 10 million untreated cells (Figure 2C).

13. Wash the cells with 1 mL cold DPBS (with protease inhibitors) one time.
14. Spin down the cells at 1,000 × *g* for 5 min at 4°C. Remove the supernatant.
15. Resuspend the cell pellets in 1 mL of lysis buffer (with protease inhibitors). Pipet up and down until the pellets are disrupted. Keep the tubes on ice for 10 min. Centrifuge the samples at 1,000 × *g* for 5 min at 4°C.

The following steps are the first restricted enzyme digestion (1^st^ RE digestion) (Figure 2D).

16. Carefully remove the supernatant and resuspend the cell nuclei with 440 μL distilled water (DNase and RNase free).
17. Add 60 μL 10× digestion buffer and 15 μL of 10% SDS.
18. Incubate the samples in a thermomixer at 37°C with 900 rpm shaking for 1 h.
19. Add additional 75 μL of 20% Triton X-100 into each sample. Pipet up and down to mix all the samples.
20. Incubate the samples in a thermomixer at 37°C with 900 rpm shaking for another 1 h.
21. Take 5 μL of each sample as “undigested control”. Save the samples in −20°C.
22. Add 200 U of first restricted enzyme (1^st^ RE) (in this case, we use DpnII.) to each sample. Pipet up and down to mix all the samples.
23. Incubate all the samples in thermomixer at 37°C with 900 rpm shaking for another 4 h.
24. Add additional 200 U first restricted enzyme (DpnII) in each sample and incubate overnight at 37°C with 900 rpm shaking.
25. Take 5 μL of digestion sample from each sample. Process the digestion efficiency quality control.
26. Add 40 μL 10 mM Tris (pH 8.0) into the “undigested controls” and “digestion samples”.
27. Add 5 μL protease K (10 mg/mL) into each sample and incubate at 65°C at least 2 h.
28. Add 8 μL DNA loading buffer into each sample and take 10 μL each sample for the DNA agarose gel (1%) as quality control (example gel in Figure 3A). Use 3 μL of 1 kb DNA ladder to mark the DNA fragments. Keep the rest of samples in −20°C for further usage.

**Figure 3:**
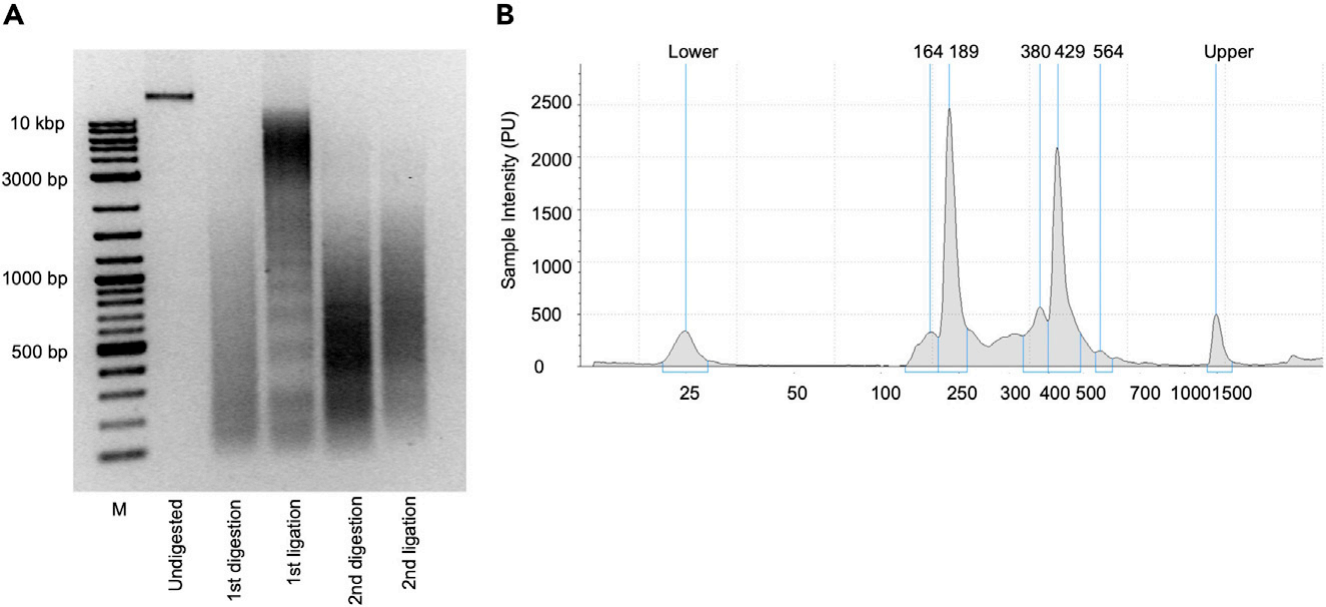
Quality control for 4C-seq (A) An example gel picture of the products from the 4C protocol. (B) TapeStation test for the quality control of the DNA distribution of the 4C-seq library.
29. If the digestion is good, the first ligation can be proceeded. Otherwise, repeat the digestion by adding additional 200 U of first enzyme (DpnII) and incubate overnight at 37°C with 900 rpm shaking.

**CRITICAL:** The ideal digestion efficiencies are somewhat different according to the enzyme selection, but the proportion of the digestion fragments should be less than 10 kb compared to the undigestion control and the distribution of the fragments are dispersed between 200 bp to 3 kb (example gel in Figure 3A).

The following steps are the first ligation (Figure 2E).

30. Heat-inactivate the first enzyme (DpnII) using 65°C for 20 min and cold down the samples on ice.
31. Transfer the samples into 50 mL tubes and add 700 μL 10× T4 ligation buffer and distilled water (DNase and RNase free) up to 7 mL.
32. Add 10 μL T4 ligase (4000 NEB units or equal to 20 Welss Units) to each sample and pipet up and down to mix all the samples.
33. Incubate all the samples at room temperature with gentle shaking overnight.
34. The next day, take 50 μL of each ligation sample and add 5 μL protease K (10 mg/mL) and incubate for at least 2 h at 65°C.
35. Run a DNA agarose gel (1%) as quality control to check the 1^st^ ligation efficiency with the previous digestion samples (step 28) (example gel in Figure 3A).
36. If the ligation quality is satisfied, process the reverse-crosslinking by adding 30 μL protease K (10 mg/mL) and incubating all the samples at 65°C overnight. Otherwise add 50 μL additional ATP (10 mM) to each sample and incubate them overnight at room temperature with gentle shaking (repeat 33–35 for re-ligation).

**CRITICAL:** When doing the ligation, it is better to avoid the cell pellets formation at the bottom, which will affect the ligation efficiency. The shaking rate should be less than 50 rpm/min.

The following steps are the first genomic DNA purification.

37. Add 30 μL RNase A (10 mg/mL) and pipet up and down to mix all the samples and incubate them for 30 min at 37°C in a water bath.
38. Add 7 mL phenol–chloroform and vigorously mix all samples using a vortex for 5 min.
39. Centrifuge all samples for 20 min at 2,000 × *g* at room temperature.
40. Carefully remove the upper aqueous phase into new 50 mL tubes.
41. Add 7 mL distilled water (DNase and RNase free), 1.5 mL NaAc (2 M, pH 5.6), 10 μL glycogen (20 mg/mL) and 35 mL 100% ethanol. Mix all tubes well.
42. Store all samples in −80°C at least one hour (the sample must be completely frozen up when processing the next step).
43. Centrifuge all samples for 20 min at 2,000 × *g* at 4°C.
44. Pump out the supernatant and wash the pellet with 10 mL of cold 70% ethanol (−20°C).
45. Centrifuge all samples for 20 min at 2,000 × *g* at 4°C.
46. Pump out the supernatant and air dry the DNA pellets at room temperature.
47. Add 450 μL 10 mM Tris (pH 8.0) to dissolve the DNA pellets and transfer the dissolved DNA into 1.5 mL tubes, which is 3C DNA template.
48. Measure the DNA concentration using Qubit. Take 5 μL samples and add 45 μL 10 mM Tris (pH 8.0) and keep them in −20°C with previous digestion and ligation samples for future usage.

∗ SAFE Stop point. The 3C DNA can be stored at −20°C for a few months.

**CRITICAL:** Do not leave the DNA pellets overly dry as it will affect DNA solubility.

The following steps are the second enzyme digestion (2^nd^ RE digestion) (Figure 2F).

49. Add 50 μL 10× second digestion buffer (in our case we used NlaIII) and 100 U NlaIII. Pipet up and down to mix all samples.
50. Incubate all samples in a thermomixer at 37°C overnight with 900 rpm shaking.
51. Take 5 μL each sample and dilute it in 45 μL 10 mM Tris (pH 8.0). Add 10 μL DNA loading buffer and take 10 μL of samples to run a DNA agarose gel (1%) as quality control to check the 2^nd^ digestion efficiency with the previous first ligation samples. Save the rest of samples with previous digestion and ligation samples for future usage.

The following steps are the second ligation (Figure 2G).

52. Heat-inactivate the second digestion enzyme using 65°C for 20 min and cold down the samples on ice.
53. Transfer all samples to 50 mL tubes and add 700 μL T4 ligation buffer (10×) and distilled water (DNase and RNase free) up to 7 mL.
54. Add 10 μL T4 ligase (4000 NEB units or equal to 20 Welss units) to each sample and pipet up and down to mix all the samples.
55. Incubate all the samples at room temperature without shaking.
56. Run a DNA agarose gel (1%) as quality control to check the 2nd ligation efficiency with the previous digestion samples (example gel in Figure 3A).

The following steps are the second genomic DNA purification.

57. Add 7 mL distilled water (DNase and RNase free), 1.5 mL NaAc (2 M, pH5.6), 10 μL glycogen (10 mg/mL) and 35 mL 100% ethanol to the second ligation sample. Mix all tubes well.
58. Store all samples in −80°C at least 1 h (the sample must be completely frozen up when processing the next step).
59. Centrifuge all samples for 20 min at 2,000 × *g* at 4°C.
60. Pump out the supernatant and wash the samples with 10 mL of cold 70% ethanol (−20°C).
61. Centrifuge all samples for 20 min at 2,000 × *g* at 4°C.
62. Pump out the supernatant and air dry the DNA pellets at room temperature.
63. Add 400 μL 10 mM Tris (pH 8.0) to dissolve the DNA pellets and transfer the dissolved DNA into 1.5 mL tubes.
64. Re-purify the samples with the QIAquick PCR purification kit following the instructions. Use four columns per sample and elute the DNA with 80 μL 10 mM Tris pH8.0 and mix the four eluted DNA samples together (in total around 320 μL DNA per mixed sample).
65. Measure the DNA concentration using Qubit or NanoDrop. Take 5 μL sample and add 45 μL 10 mM Tris and keep them in −20°C with previous digestion and ligation samples for future usage. At this point, the 4C template preparation is done and the sample could be visualized using a DNA agarose gel (1%) with all proceed DNA samples in the previous steps (example gel in Figure 3A). In mouse macrophages, the concentration of 4C template DNA after the last purification is 50–100 ng/μL. The 4C template DNA samples can be stored in −20°C or come to the 4C-PCR step directly. For reducing 4C library bias and increasing library complexity, at least 8 PCR need to be performed and pooled for one bait primer pair.

∗ SAFE Stop point. The DNA can be stored at −20°C for a few months.

### 4C-PCR to enrich the DNA interaction by the viewpoint primer

**Timing: 1–2 days**

66. Set 8 independent PCR reactions for one 4C template sample to avoid the amplification bias, and each reaction is prepared as follows:

**Table:** 

Reagent	Amount
Reading primer (20 *μ*M Stock)	5 μL
Inverse primer (20 *μ*M Stock)	5 μL
10× PCR buffer 1	5 μL
dNTP (10 mM)	1 μL
200 ng of 4C template	Accordingly
Expand Long Template Polymerase	0.35 μL
ddH_2_O	Accordingly
Total	50 μL

67. PCR amplification program is applied as follows: 94°C for 2 min; 94°C for 10 s, 55°C 1 min, 68°C 3 min, apply for 30 cycles; 68°C 5 min.
68. Clean up the 4C-PCR products with ChargeSwitch PCR Clean-Up Kit to remove the primers following the manufacturer’s instruction. Elute the DNA in 50 μL 10 mM Tris pH 8.0. Pool the PCR samples together and measure the DNA concentration using Qubit Assay kit.
69. Run a DNA agarose gel (1%) with 10 μL purified PCR products together with the previous second ligation samples to check the amplification quality. Then the 4C-PCR products can be used for the sequencing library preparation.

**CRITICAL:** 4C-PCR (inverse PCR) primer design principle is by previous publication, and it is optimized before the experiment (Krijger et al., 2020; van de Werken et al., 2012). The reading primer (within 20 bp to the first restriction enzyme site) and the reverse primer (within 100 bp to the second restriction enzyme site). In our case, we selected DpnII (first enzyme) and NlaIII (second enzyme). The primer is needed to test the specificity before the real 4C-PCR. A pre-PCR is preferably needed to determine the amplification sensitivity due to the 4C template concentration. A good 4C-PCR will give a strong DNA smear with “self-ligation” and “undigested” bands and different with the 4C template distribution (Figure 3B).

### Library preparation for sequencing

**Timing: 2–3 h**

The library preparation of 4C-seq was performed directly using Takara ThruPLEX DNA-Seq kit according to the manufacturer’s instructions. Similar commercial kits such as NEXTFLEX Rapid DNA-Seq Library Prep Kit (PerkinElmer, NOVA-5144-01) and NEBNext Ultra II DNA Library Prep Kit for Illumina (New England BioLabs, E7103) can be used for the library preparation according to the instructions.

70. DNA repair and template preparation. Mix 10 μL of the 4C-PCR products with 2 μL template preparation buffer and 1 μL preparation enzyme. Pipet up and down and put the samples in a PCR machine. Incubate the sample at 25°C 25 min, 55°C 20 min. Total DNA amount need to be considered as it effects the PCR cycles in step 72. Follow the suggestions from the manufacturer’s instructions.
71. Stem-loop adapter assemble and library synthesis. Add 1 μL of the library synthesis buffer and 1 μL library synthesis enzyme in the samples and incubate at 22°C 40 min.
72. Add index primer and library amplification. Add 25 μL library amplification buffer, 4 μL distilled water and 1 μL library amplification enzyme.
73. Add the individual index which provided from the Takara ThruPLEX index kit. Set up the PCR amplification program as (5–8 PCR cycles are recommended):

**Table:** 

Steps	Temperature	Time	Cycles
Extension	72°C	3 min	1
Cleavage	85°C	2 min	1
Denaturation	98°C	2 min	1
Addition of indexes	98°C	20 s	4
	67°C	20 s	
	72°C	40 s	
Amplification	98°C	20 s	5–16 cycles, dependent on the starting DNA
	72°C	50 s	
Hold	4°C	forever

74. After the PCR amplification, take 1 μL PCR sample to check the DNA concentration using Qubit. If the DNA concentration was between 10–30 ng/μL, the DNA purification could be processed in the next step. If the DNA concentration in less than 5 ng/μL, additional cycles are needed (normally two cycles). It is not recommended to over amplify the sample.

The following steps are the library purification using AMPure beads (double selection strategy) (Figure 2H).

75. Add 50 μL of library sample into a 96 well plates and add 25 μL AMPure beads (0.5×). Mix well using pipets at least ten times.
76. Incubate 5 min at room temperature.
77. Put the plate on the magnetic Stand-96 for 5 min.
78. Transfer the supernatant into a new 96 well and not touch the beads (to remove the big fragment).
79. Add 60 μL AMPure beads (1.2×) again in the supernatant and mix well.
80. Incubate 5 min at room temperature.
81. Put the plate again on the magnetic Stand-96 for 5 min.
82. Transfer the supernatant into a new 96 well and not touch the beads (to remove the index primers).
83. Wash the beads with 200 μL fresh 80% ethanol for 30 s.
84. Remove the supernatant and add 200 μL fresh 80% ethanol for another 30 s.
85. Remove the supernatant carefully and air dry the beads for 2–5 min at room temperature.
86. Add 35 μL distilled water and pipet up and down to mix the beads well.
87. Incubate 5 min at room temperature.
88. Put the plate again on the magnetic Stand-96 for 5 min.
89. Collect 32 μL of the supernatant with a 1.5 mL tube.
90. Measure the DNA concentration using Qubit Assay kit.
91. Use TapeStation system to check the 4C-seq library quality. After AMPure beads selection, there should be no primer dimers and big fragments left. For an example of 4C library distribution, please refer to Figure 3B.
92. Once the 4C-seq samples pass the quality control (TapeStation, Figure 3B), it is ready for the sequencing. Mix all 4C-seq library samples with appropriate concentration.

**CRITICAL:** 4C-seq only requires 1–5 million reads for the downstream analysis. Different 4C-seq samples have different index sequences which can be demultiplexed with the indexes even they share the same reading primer. Ideally, samples with different treatments will make the comparison more sense as the DNA interaction is related to the gene expression. For more details, please refer to (Huang et al., 2021). Single read output and 50 bp or 75 bp read depth can be used for the Illumina sequencing. In our case, we used 75 bp reads output.

### Data processing

**Timing: 2–3 days**

**Figure 4:**
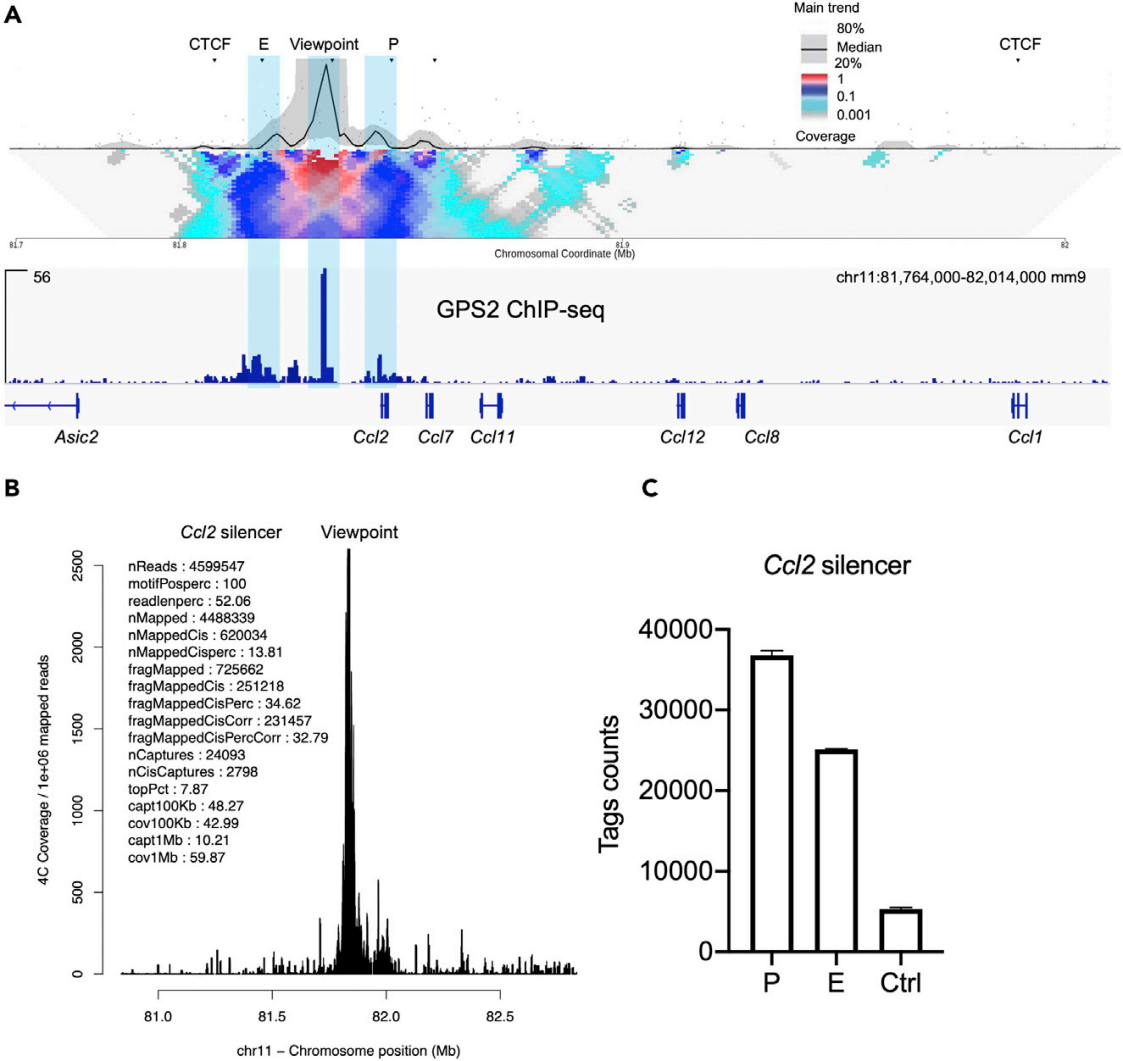
Data visualization for 4C-seq (A) Heatmap showing the 4C contacts from the silencer bait of *Ccl2* and the integration with the ChIP-seq data (GPS2). The CTCF sites, enhancer and promoter regions are highlighted. (B) Normalized 4C-seq coverage profiles within the *Ccl2* gene locus. The reads are normalized 1e+10^6^ total reads and the mapping statistic within *Ccl2* locus are shown on the left panel. (C) Tag counts showing the *Ccl2* enhancer (E), promoter (P) and control regions (Ctrl) by *Ccl2* silencer bait. Data are represented as mean ± SEM.

In this section, we used two different strategies to perform the 4C-seq data analysis (Figure 4).

93. Pre-analysis: Before the analysis, the FASTQ sequence quality is inspected by running “fastqc ∗.fastq.gz” according to routines.
94. Pre-analysis: Trim the FASTQ files to remove short reads using:

>homerTools trim -min 70 ∗.fastq

**First analysis:** Relatively interaction frequency analysis using 4Cseqpipe (Figure 4A) (van de Werken et al., 2012).

95. Download the 4Cseqpipe (https://github.com/changegene/4Cseqpipe) in Linux systems first. Follow the example and fill the “index.txt” file in “rawdata” folder with the experiment designs. In our case, we used DpnII (first enzyme) and NlaIII (second enzyme) to make the 4C-seq samples. We selected *Ccl2* silencer region as viewpoint (VP) to visualize the DNA interaction. So, in the “index.txt” file we filled with the DNA coordinates with chr11: 81835725 (*Ccl2* silencer), see below. Create a gene feature file for the gene of interest with ChIP-seq binding, gene promoter, CTCF or cohesion and any other gene coordinates for visualization.

**Table:** 

Id	run	lane_no	Exp	primer_seq	spcies_name	first_cutter	first_cutter_seq	sec_cutter_name	sec_cutter_seq	linearization_name	linearization_seq	bait_chromo	bait_coord	seq_len	raw_fname
1	2018_12	0	RAW_4C_seq_silencer_rp1	GTAAAAG TGATTAG AAGAGA GATC	Mus_musculus	DpnII	GATC	NlaIII	CATG	NA	NA	11	8183 5725	40	RAW_4C_seq_silencer_rp1.fastq.gz
2	2018_12	0	RAW_4C_seq_silencer_rp2	GTAAAA GTGATTA GAAGAGA GATC	Mus_musculus	DpnII	GATC	NlaIII	CATG	NA	NA	11	8183 5725	40	RAW_4C_seq_silencer_rp2.fastq.gz

96. Build the enzyme digested genome with the selected combination using: perl 4cseqpipe.pl –build_re_db –first_cutter XXXX –second_cutters YYYY –trackdb_root /root_directory. In this case, we use DpnII (GATC, first cutter) and NalIII (CATG, second cutter) combination to build the fragment library. So, the code in the terminal is:

>perl 4cseqpipe.pl –build_re_db –first_cutter GATC –second_cutters CATG –trackdb_root /root_directory

97. Extract the FASTQ file into 4Cseqpipe by running:

>perl 4cseqpipe.pl –fastq2raw –ids 1 –rawdir directory –fastq_fn RAW_4C_seq_silencer_rp1.fastq

98. Align the 4C-Seq products to digested genome by running:

>perl 4cseqpipe.pl –map –ids 1

99. Plot the interaction results by running:

>perl 4cseqpipe.pl -nearcis -calc_from 81774000 -calc_to 81974000 -stat_type median -trend_resolution 2000 - figure_fn $1_$2_200kb.png -feat_tab rawdata/ccl2_feature.txt

**Second analysis:** Compare the interaction strength using pipe4C (Figure 4B) (Krijger et al., 2020).

100. Download and install the pipe4C (https://github.com/deLaatLab/pipe4C) in UNIX or macOS systems first.
101. Fill the “VPinfo.txt” file with the designed 4C information of interest as below:

**Table:** 

expname	spacer	primer	firstenzyme	Secondenzyme	genome	vpchr	vppos	analysis	fastq
RAW_4C_seq_silencer_rp1_cis	0	GTAAAAG TGATTA GAAGAG AGATC	DpnII	NlaIII	mm9	11	81835725	cis	RAW_4C_seq_silencer_rp1.fastq.gz
RAW_4C_seq_silencer_rp2_cis	0	GTAAAAG TGATTA GAAGAG AGATC	DpnII	NlaIII	mm9	11	81835725	cis	RAW_4C_seq_silencer_rp2.fastq.gz
RAW_4C_seq_silencer_rp1_all	0	GTAAAAG TGATTA GAAGAG AGATC	DpnII	NlaIII	mm9	11	81835725	all	RAW_4C_seq_silencer_rp1.fastq.gz
RAW_4C_seq_silencer_rp2_all	0	GTAAAAG TGATTA GAAGAG AGATC	DpnII	NlaIII	mm9	11	81835725	all	RAW_4C_seq_silencer_rp2.fastq.gz

102. Update the “conf.yml” file with the correct Bowtie2 index location and the enzyme combinations selected in the experiment. Indicate the working FASTQ file location. As the default setting, pipe4C program will use 1 M total tag counts to normalize all 4C-seq samples which make the interaction strength comparison possible.
103. Set the root dir within the “conf.yml” folder and run the R script as following:

>Rscript pipe4C.R --vpFile=indicated_dir/VPinfo.txt --fqFolder=/ indicated_dir/FASTQ_file/ --outFolder=/ indicated_dir /outF/ --cores 8 --plot –wig

104. Once the program finish, upload the ‘.wig’ files to Galaxy (https://usegalaxy.org/) and use the “Wiggle-to-Interval converter” to change the file to ‘. Interval’ files. Download the ‘. Interval’ files and change the file format to ‘.txt’. Add the specific head to the column with ‘chr start end stand tags’. Import the files to R, and execute the code to extract the tags of target regions:

>Ccl2_silencer<-read.delim2("RAW_4C_seq_silencer_rp1_WIN21.txt",head=TRUE)

>Ccl2_silencer_f<-subset(Ccl2_silencer, start >81816382 & start <81822382)

>sum(Ccl2_silencer_f$tags)

In our case, we used *Ccl2* promoter and enhancer 4C bait to find the potential interaction during gene activation. We used chr11: 81,816,382–81,822,382 (*Ccl2* enhancer), chr11:81,846,000–81,852,000 (*Ccl2* promoter) and chr11: 81,785,479–81,791,479 (Control region) to count the tags.

105. Visualize the tags in GraphPad software (Figure 4C). If three biology replicates are provided, the statistical analysis could be done. Otherwise, show the results with ± SEM plots.

***Note:*** The 4Cseqpipe provides relative interaction frequency plots around the viewpoint to display the potential interaction regions. This method uses viewpoint as the centre (treat as 1). The captured 4C contacts will be plotted according to the relative frequency. The pipe4C provides the quantitative normalization files, which use the total tags counts (10^6^) and the target chromosome region as background.

**CRITICAL:** DNA interaction ranges are different from gene to gene which are dependent on the local DNA environment, TF binding, histone modification and CTCF location. So, it is better to make multiple comparisons using different ChIP-seq data and to use many plot ranges from 100 kb to 1 mb. It is useful to find all the possible *cis* interaction regions (Figure 4A). On one hand, the ChIP-seq data will give the TFs and co-regulators binding center which participate in the DNA loop formation. On the other hand, some gene activation histone marker ChIP-seq (H3K27ac, H3K4me3, etc.) will give the epigenetic environment changes, which will indicate the 4C-seq changes make any sense. For more details, please refer to (Huang et al., 2021). In our case we do not check the *trans* interaction search since it is not common and needs additional primer designs.

## Expected outcomes

### DNA fragmentation during the 4C template preparation

The macrophage genome will be reorganized by the enzyme digestion and ligation through the whole 4C protocol. In the first enzyme (DpnII) digestion and first ligation process, the distant enhancer and promoter interactions will be formed which are called the 3C templates. Normally, big DNA fragments will be formed during the first ligation which is critical for the success of the 4C experiment. In the second enzyme (NlaIII) digestion and second ligation process, the 3C templates were digested and small circular DNA will be formed in this step. In the 4C-PCR step, all interaction of specific region of interest (*Ccl2* promoter) will be amplified for the later sequencing step. DNA gels will be frequently used as quality control throughout the whole 4C process (Figure 3A). One example of DNA distribution (TapeStation) is shown in Figure 3A with the 4C protocol. It is highly recommended to analyze the DNA distribution (TapeStation) before the Illumina sequencing. Normally, the small and large size of genomic DNA will be removed during the clean-up steps (Figure 3B). Indexes contamination is an important issue for high quality sequencing output. Re-doing the size selection is recommended if there are small fragments (<100 bp) in the sample.

### Basic reads information

When processing the pre-analysis using fastqc, overall sequencing quality and the sequence distribution will be shown. Normally, we will remove the short reads less than 70 bp. The reads before and after trimming are shown in Table 1. The related mapping statistics are shown in Table 2 and the Figure 4B for the individual package.

**Table 1:** Summarized statistic results from HOMER

Statistics	Replicate 1	Replicate 2
Total reads	10384078	10784319
After trimed (< 70 bp)	8681704	8241039

**Table 2:** Mapping statistics by 4Cseqpipe

	Replicate 1	Replicate 2
Reading primer reads	4000313	3744352
Mapped reads	886866	867499
Ignored reads	2893595	2662672
Low qual reads	221115	213423
Cis reads vs. tot	0.350112	0.349505

### Interaction frequency plot

The 4Cseqpipe will generate a relatively plot using the viewpoint region. It is recommended to make several plots with different DNA ranges. We used 100 kb, 200 kb, 1 mb viewpoint windows to check the interaction within *Ccl2* locus. The 4C interactions can also be integrated together with transcription factor and coregulator ChIP-seq data such as CTCF (insulating factors), JunB and corepressor complex (GPS2 etc.), as the transcription factor complexes facilitate the loop formation and dynamics (Figure 4A). The 4C-seq detects loop changing dynamics by different treatments can also be co-analyzed with gene expression for better understanding the regulatory mechanisms in the chromatin level.

### Normalized tag counts by specific regions of interests

Using ‘pipe4C’, we can get a coverage plot with the normalized data to display all the fragments within the target region, which gives an overview of the 4C experiment (Figure 4B). The ‘pipe4C’ can also generate wig file output which can be used to normalize the data with total tag counts. It is useful to compare the interaction strength in any element region marked by transcription factor (Figure 4C). In our case, we used *Ccl2* silencer bait to check the loops and qualify the interaction tags strength within *Ccl2* enhancer, promoter, and control regions. If condition permits, the statistical analysis could be done in this step, otherwise the internal control region is needed to show the loop changes.

## Limitations

Far-*cis* (> 5 Mb) and *trans* interactions are rare in genome. Because of the low efficiency, the reproducibility of such interactions is low in 4C-seq. We ignored such type of DNA interaction search in macrophages. Hi-C is recommended to catch such distal interactions.

The DNA interaction frequency by the viewpoint primer is largely depending on the distribution of the first enzyme in the target regions of interest. This will possibly cause a shift of the interaction from the TF binding center. Therefore, the cistrome or epigenome datasets (histone and key TF ChIP-seq) should be included to make a solid conclusion of the core interaction regions, especially for motif analysis.

Compared to Hi-C, 4C-seq is a low throughput method which provides only the interested regions’ binding profiles. In contrast, it provides relatively high-resolution results which might help us to know the details within the gene locus. The combination of Hi-C and 4C-seq could be a good experimental strategy to overcome the genome-wide scale and the resolution.

The restriction enzyme bias in 4C-seq. The 4C-seq relies on the location of the first restriction enzyme. If the genomic location lacks the commonly used enzymes, it will be difficult to get DNA interaction profiles.

## Troubleshooting

### Problem 1

Low efficiency in first digestion (first enzyme, step 28).

The first restrict enzyme digestion and ligation is the crucial steps as they form the 3C contacts. It is highly recommended to determine the digestion efficiency before and after the first enzyme incubation before further processing the DNA.

### Potential solution

Design two pair of primers to amplify the genomic region of interest which contains the reading primer. One pair of primers (test primer), which spans the first enzyme site of the reading primer region (including the reading primer itself) is used for checking the digestion efficiency. The other one (control primer) which does not amplify any first enzyme region, serves as an internal control. Additional steps can be used as follows:

• Take 5 μL of each sample (before and after first digestion) and add 45 μL 10 mM Tris (pH 8.0).
• Add 5 μL protease K (10 mg/mL) in each sample. Mix well and incubate at 65°C for 1 h.
• Add 5 μL RNase (10 mg/mL) in each sample. Mix well and incubate at 37°C for 20 min.
• Purify the genomic DNA using QIAwave DNA Blood & Tissue kit.
• Use 50 μL 10 mM Tris (pH 8.0) to elute the DNA.
• Use 2 μL of DNA sample and set up a PCR system to check the digestion efficiency. The Ct value of the internal control primer between “un-digestion” and “digestion” sample is used as normalize factor. The relatively cut efficiency is calculated by the ΔCt value using the test primer. Good first digestion efficiency is more than 90%.

While there are many factors that can affect the digestion efficiency including insufficient amount of enzyme, incomplete cell lysis and over crosslinking. These above factors are needed to be taken into consideration if the digestion is insufficient.

### Problem 2

Digestion distribution (both first and second enzyme, steps 28 and 51).

Different cell types have different chromatin contacts which make the first and second enzymes digestion vary even with the same enzyme combinations. Therefore, it is hard to directly compare with the DNA gel from this protocol among different cell types.

### Potential solution

Make a pre-experiment for a whole 4C protocol including the 4C-PCR step, which will give a general idea of the digestion profile in the target cell type.

### Problem 3

Low ligation efficiency (both first and second ligation, steps 35 and 56).

### Potential solution

The detergent in the buffer is usually the reason that hampers the ligation process. Make sure to dilute the detergent concentration during the ligation process. Also increase the incubation time and ligase amount for unsuccessful ligation.

## Resource availability

### Lead contact

Further information and requests for resources and reagents should be directed to and will be fulfilled by the lead contact, Zhiqiang Huang (zhiqiang.huang@ki.se).

### Materials availability

All reagents, oligonucleotides, sequencing data and software are listed in the key resources table.

## Data Availability

4C-seq data in this study have been deposited in NCBI’s Gene Expression Omnibus and are accessible through GEO series accession number GSE195943. The related 4C-seq dataset is GSE130383.
